# “Strike Early and Strike Strong”: Low LDL-Cholesterol and Low Albumin Predict Statin Hyporesponsiveness in Acute Coronary Syndrome

**DOI:** 10.3390/jcm15062312

**Published:** 2026-03-18

**Authors:** Şükriye Uslu, Gülsüm Meral Yılmaz Öztekin

**Affiliations:** Department of Cardiology, Health Science University, Antalya Training and Research Hospital, 07100 Antalya, Turkey; gmeralyilmaz@gmail.com

**Keywords:** statin response, hyporesponsiveness, lipid-lowering therapy, ≥50% LDL-C reduction in LDL-C, albumin, acute coronary syndrome

## Abstract

**Background/Objectives:** Many patients with acute coronary syndrome (ACS) fail to achieve adequate low-density lipoprotein-cholesterol (LDL-C) reduction, despite receiving high-intensity statin therapy. Identifying patients requiring early combination therapy remains a challenging task. This study aimed to determine the prevalence of statin hyporesponsiveness in patients with ACS and investigate the predictive role of baseline LDL-C and albumin levels. **Methods:** This retrospective study enrolled 366 patients with ACS treated with high-intensity statins (atorvastatin 40–80 mg). Hyporesponsiveness was defined as LDL-C reduction of <50% at 21–28 d. The baseline parameters were analyzed using logistic regression and receiver operating characteristic (ROC) curve analysis. **Results:** Hyporesponsiveness was observed in 63.1% of patients. Hyporesponders had significantly lower baseline albumin (41.7 vs. 43.3 g/L, *p* = 0.0002) and LDL-C (126.6 vs. 147.3 mg/dL, *p* < 0.0001) levels. Categorical analysis revealed that the combination of baseline LDL-C < 100 mg/dL and albumin < 40 g/L predicted hyporesponsiveness in 95.7% of cases. ROC curve analysis identified optimal predictive cut-offs of 128.50 mg/dL for LDL-C (area under the curve (AUC): 0.652) and 41.15 g/L for albumin (AUC: 0.618). The combined LDL-C + albumin model demonstrated superior predictive performance with an AUC of 0.670 (95% CI: 0.615–0.725). **Conclusions:** Low baseline LDL-C and low albumin are strong predictors of statin hyporesponsiveness in patients with ACS. These routinely obtained biomarkers can identify very high-risk patients who may benefit from proactive combination lipid-lowering therapy from hospital discharge, supporting the “strike early and strike strong” strategy and challenging the traditional stepwise approach.

## 1. Introduction

Cardiovascular disease (CVD), particularly ischemic heart disease and stroke, remains the leading cause of morbidity and mortality globally. Acute coronary syndrome (ACS), the most critical manifestation of ischemic heart disease, requires early and aggressive treatment strategies [1,2]. Despite hyperlipidemia being a modifiable risk factor for atherosclerotic cardiovascular disease (ASCVD) and the existence of clinical guidelines and evidence supporting the benefits of lipid-lowering therapy (LLT), real-world data reveal a significant treatment gap in the management of dyslipidemia [3]. Long-standing studies have shown that high-density lipid-lowering therapy (LLT) improves the prognosis after acute coronary syndrome (ACS); this concept is also supported by the principle of “the lower the better” in reducing low-density lipoprotein cholesterol (LDL-C) [4,5]. The landmark IMPROVE-IT trial, published in 2015, revealed that lower LDL-C levels achieved by adding ezetimibe to statin therapy elicited greater cardiovascular benefit compared with statin monotherapy [6].

In light of this evidence, the “strike early and strike strong” approach was proposed in 2022 by the European Society of Cardiology working groups, recommending the initiation of combination LLT using high-intensity statins and ezetimibe in the early period after ACS [7]. This approach has been strongly supported by the SWEDEHEART registry data, which revealed that early and sustained lowering of non-high-density lipoprotein cholesterol (non-HDL-C) following ACS is associated with the best prognosis, and it has been argued that the traditional stepwise approach leads to potentially harmful delays [8]. The current guidelines recommend early initiation of statin and ezetimibe combination therapy [2,9,10].

Moreover, the guidelines recommend achieving an absolute LDL-C target (<55 mg/dL) along with at least 50% reduction from baseline in very high-risk patients [2]. However, the clinical importance of the equivalence of this dual target has been questioned by a recent study. Fujioka et al. showed that achieving ≥50% LDL-C reduction in LDL-C levels was superior to reaching the absolute LDL-C target of 55 mg/dL in reducing the rates of major adverse cardiovascular events (MACE) [11]. This crucial finding indicates that hyporesponsiveness, defined as an inadequate response to statin therapy, may be a stronger and more clinically meaningful prognostic marker than achieving a specific LDL-C value.

Identifying the determinants underlying hyporesponsiveness is crucial, owing to its clinical significance in predicting cardiovascular outcomes. In recent years, low albumin levels have been shown to be associated with poor prognosis [12,13]. Albumin serves as an indicator of nutritional status and as an important inflammatory marker, while also interacting with cholesterol metabolism [12,13]. Moreover, baseline LDL-C level is an important determinant of the statin response [11,12,13,14]. These observations indicate that both albumin and baseline LDL-C may serve as independent predictors of statin hyporesponsiveness.

Determining patients who would benefit most from the ‘strike early and strike strong’ strategy is crucial. Therefore, this study aimed to assess the prevalence of statin hyporesponsiveness after ACS and investigate the role of baseline LDL-C and albumin levels in predicting the statin response.

## 2. Materials and Methods

### 2.1. Study Population

We retrospectively analyzed 712 patients diagnosed with unstable angina pectoris (USAP), non-ST-elevation myocardial infarction (non-STEMI), and ST-elevation myocardial infarction (STEMI) at a tertiary hospital between January and December 2024. The Academic Ethics Committee of the University of Health Sciences, Antalya Training and Research Hospital, approved this retrospective study and waived the requirement for informed consent (2025-460). All procedures were conducted in accordance with the Declaration of Helsinki. The exclusion criteria were-as follow: patients not requiring percutaneous coronary intervention (PCI, *n* = 17); patients with a history of revascularization [PCI, *n* = 140; coronary artery bypass graft (CABG, *n* = 24)]; in-hospital death (*n* = 17); patients transferred to cardiovascular surgery and other departments with a plan for CABG (*n* = 16); patients who did not attend the third-week LDL-C follow-up (*n* = 113); patients prescribed antibiotics (*n* = 6); patients with alanine transaminase values more than three times the upper limit (ALT × 3, *n* = 4); and patients with a glomerular filtration rate (GFR) <30 mL/min/1.73 m^2^ (*n* = 9). In total, 366 patients who were not taking statins before their ACS diagnosis and who continued high-intensity statin therapy initiated after their ACS diagnosis were included in our study. We recorded LDL-C values measured during admission to the coronary intensive care unit and at the first outpatient follow-up (21–28 days) after discharge. We classified patients as hyporesponders if they achieved <50% LDL-C reduction from baseline and as responders if they attained ≥50% LDL-C reduction. High-intensity statin therapy was defined as monotherapy with atorvastatin at doses of 40 mg or 80 mg.

### 2.2. Blood and Lipid Measurements

Blood samples were obtained from the initial blood draws at coronary intensive care unit admission. We analyzed the biochemical and lipid parameters using the Beckman Coulter AU5800 (Beckman Coulter Inc., San Jose, CA, USA), and the complete blood count using the Sysmex XT-2000i (Sysmex, Kobe, Japan). LDL-C levels were calculated using the Friedewald formula, except in cases where the triglyceride levels were >400 mg/dL [15].

### 2.3. Statistical Methods

All statistical analyses were performed using the SAS v. 9.4 (SAS Institute, Cary, NC, USA) software package. Continuous variables in the patient dataset were presented as the mean and standard deviation (SD) or median and interquartile ranges, while categorical variables were described as numbers and percentages. The Shapiro–Wilk test was used to determine normality of the distribution of the continuous variables. Statistical comparisons between the two groups were performed using the independent Student t-test for normally distributed variables and the Mann–Whitney U test for non-normally distributed variables. The chi-square test or Fisher’s exact test was used to analyze the relationship between two categorical variables.

Receiver operating characteristic (ROC) curve analysis was performed to determine the optimal cut-off values for albumin, LDL-C and LDL-C + Albumin, to calculate the sensitivity and specificity values, and to determine the area under the ROC curve (AUC). The point with the largest Youden index (sensitivity + specificity − 1) was selected as the optimal cut-off point. The AUC was used to compare the predictive performance of albumin and LDL-C.

Univariate binary logistic regression analysis was performed to investigate the relationship between baseline demographic, clinical, and laboratory variables and the response status (hyporesponder or responder), followed by multivariate adjustments to identify independent predictors. The final multivariate logistic regression model was used to estimate the odds ratios (OR) and their corresponding 95% confidence intervals (CI).

A significance level of 0.05 was accepted throughout the study.

Bootstrap validation was performed with 500 resamplings using SAS/STAT^®^ v. 9.4 procedures (PROC PLM RESTORE) for model validation.

## 3. Results

### 3.1. Patient Characteristics and Statin Response Outcomes

The participants’ clinical characteristics are shown in Table 1. The mean age of the 366 patients included in the study was 58.7 years, and 84.4% were men. PCI was performed for STEMI in 59.8% of participants, non-STEMI in 36.3%, and USAP in 3.8% of participants. After high-intensity statin therapy, 231 patients (63.1%) were classified as hyporesponders. The classic cardiovascular risk factors, such as diabetes mellitus, hypertension, and smoking, did not differ (statistically) significantly between the two groups (*p* > 0.05).

The albumin (41.7 vs. 43.3 g/L, *p* = 0.0002) and hemoglobin (14.2 vs. 14.8 g/dL, *p* = 0.0016) levels were significantly lower in the hyporesponder group compared with the responder group. Similarly, the total cholesterol (206.8 vs. 227.6 mg/dL, *p* < 0.0001) and LDL-C (126.6 vs. 147.3 mg/dL, *p* < 0.0001) levels were also significantly lower in the hyporesponder group.

### 3.2. Association of LDL-C and Albumin Levels with Hyporesponsiveness

When patients were categorized according to the baseline LDL-C and albumin levels, a cumulative (additive) effect of these two parameters on hyporesponsiveness emerged (Table 2). Notably, patients with baseline LDL-C <100 mg/dL and albumin <40 g/L exhibited a hyporesponsiveness rate of 95.7%, revealing a nearly complete lack of response. In contrast, the hyporesponsiveness rate decreased to 47.6% in the group with LDL-C > 160 mg/dL and albumin > 44 g/L.

**Figure 1:**
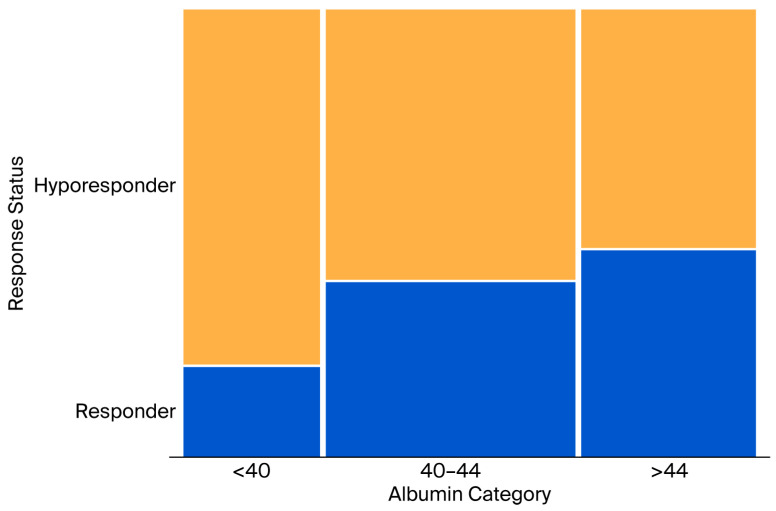
Distribution of Response Status by Albumin Levels (g/L) (Hyporesponder: less than 50% reduction from baseline LDL-C, Responder: 50% or more reduction from baseline LDL-C).

**Figure 2:**
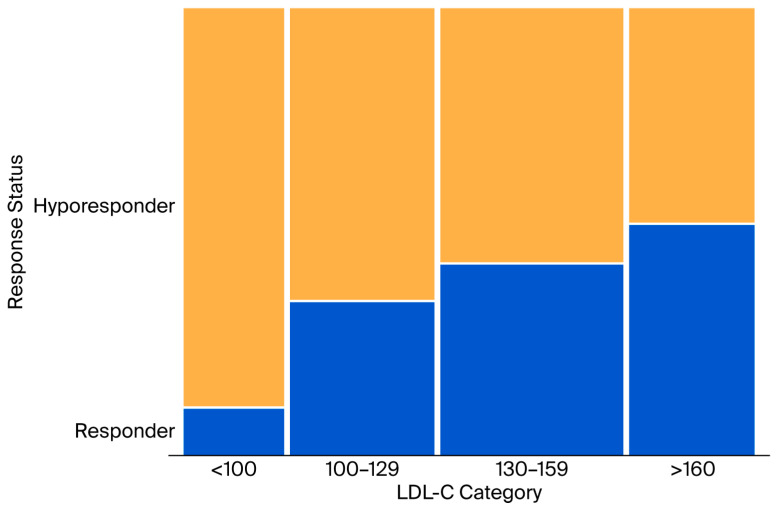
Distribution of response status by LDL-C levels (mg/dL) (Hyporesponder: less than 50% reduction from baseline LDL-C, responder: 50% or more reduction from baseline LDL-C).

Cochran–Armitage trend analysis confirmed a statistically significant increase in the proportion of patients responding to statin therapy in both the increasing albumin categories (*p* = 0.0004) and increasing LDL-C categories (*p* < 0.0001; Table 3). These trends are also visually presented in the mosaic plots in Figure 1 and Figure 2.

### 3.3. Independent Predictors of Hyporesponsiveness

Correlation analysis performed to evaluate the relationship between statin response status and variables revealed that both albumin (Somers’ D = 0.217) and LDL-C (Somers’ D = 0.308) showed a significant positive correlation with the statin response (Table 4).

In the univariate logistic regression analysis, albumin, hemoglobin, total cholesterol, and LDL-C were determined to be associated with hyporesponsiveness. However, multivariate logistic regression analysis identified baseline LDL-C (OR: 1.0, 95% CI: 1.0–1.0, *p* = 0.0041) and albumin (OR: 1.1, 95% CI: 1.0–1.2, *p* = 0.0191) as independent predictors of the statin response (Table 5).

**Figure 3:**
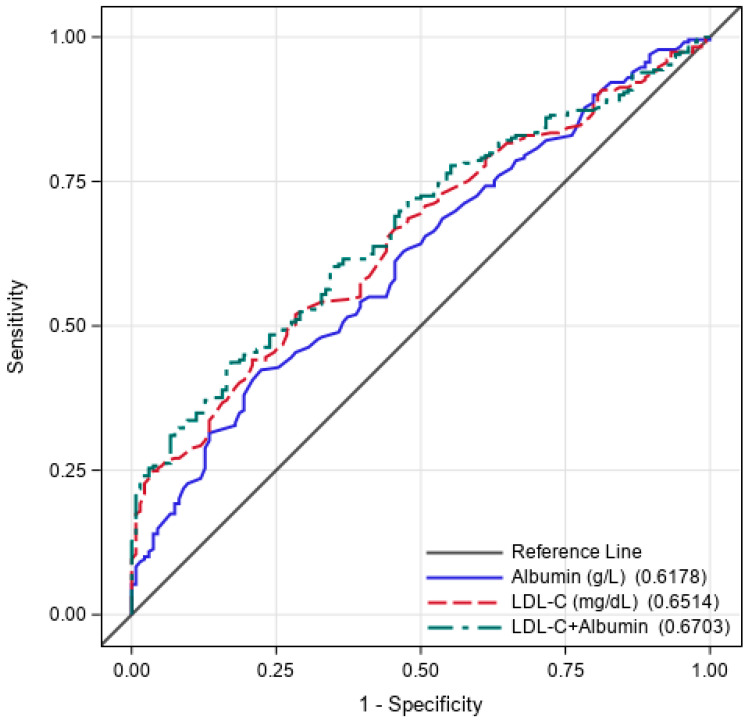
Receiver operating characteristic curve for albumin, LDL-C, and LDL-C + albumin.

ROC curve analysis revealed that the optimal cut-off values for predicting hyporesponsiveness were 41.15 g/L for albumin (AUC: 0.618, 77.6% sensitivity, 42.4% specificity) and 128.50 mg/dL for LDL-C (AUC: 0.652, 71.9% sensitivity, 51.9% specificity) (Table 6, Figure 3). When ROC analysis was performed for LDL-C + albumin, the AUC value was 0.670 ([95% CI 0.615–0.725], *p* < 0.001), which was found to be higher than albumin or LDL-C alone.

The pair-wise comparison of the AUCs of Albumin, LDL-C, and LDL-C + albumin is presented in Table 7. As can be seen from this table, LDL-C + albumin performs better than Albumin and LDL-C; the other pair-wise comparisons do not indicate any statistical significance.

### 3.4. Model Validation

The analysis yielded an expected optimism of 0.014 for the c-statistic (AUC), with optimism-corrected c-statistic values of 0.681 (original) and 0.667 (corrected), respectively. The minimal difference between the original and optimism-corrected AUC values (0.003) indicates that the model fits the data well and is robust against overfitting, demonstrating good discriminative performance of the combined model.

## 4. Discussion

The most important finding of our study, aimed at identifying which patients would benefit most critically from the “strike early and strike strong” strategy, is that 63.1% of patients receiving high-intensity statin therapy after ACS exhibited hyporesponsiveness (<50% LDL-C reduction). More importantly, this study reveals for the first time that the combination of low baseline LDL-C (<100 mg/dL) and low albumin (<40 g/L) is a novel and powerful clinical marker predicting almost complete hyporesponsiveness to high-intensity statins (95.7%). Baseline LDL-C and albumin levels were shown to have a cumulative (additive) effect on hyporesponsiveness. This finding highlights the importance of the cumulative effect of simple biochemical parameters in predicting the statin response.

Studies have used disparate definitions of statin hyporesponsiveness. Sun et al. defined it as <30% LDL-C reduction, despite moderate- and high-intensity statin use, while Tsuda et al. used the criterion of <15% LDL-C reduction after acute myocardial infarction [16,17]. In this study, patients who failed to achieve the ≥50% LDL-C reduction target recommended by the current guidelines were defined as hyporesponders [2].

Fujioka et al. reported that a <50% reduction in LDL-C carries a greater risk of cardiovascular events than that associated with failure to achieve the absolute LDL-C target (55 mg/dL) [11]. Similarly, Amarenco et al. revealed that patients with ischemic stroke who achieved LDL-C < 70 mg/dL experienced a reduction in major vascular events only when >50% LDL-C reduction was achieved, whereas no significant benefit was observed in patients achieving <50% reduction [18]. These findings support the notion that optimal therapy should entail achieving the target LDL-C as well as at least a 50% relative reduction to optimize risk reduction [11].

The clinical significance of statin hyporesponsiveness is not limited to failure to achieve target LDL-C levels. Kataoka et al. analyzed intravascular ultrasound data from 7 prospective atherosclerosis progression/regression studies, reporting that statin hyporesponders (<15% LDL-C reduction) had significantly lower baseline LDL-C levels than responders and, more importantly, these hyporesponder patients exhibited significantly greater atheroma progression [19]. These findings show that statin hyporesponsiveness is not merely a biochemical non-response but also has significant clinical implications in terms of atherosclerosis progression and cardiovascular event risk.

Albumin’s negative association with coronary artery disease may reflect its capacity to shuttle free cholesterol among various receptors, thereby facilitating the movement of free cholesterol along concentration gradients between the multiple cholesterol pools in plasma and tissues while promoting the restoration of steady-state metabolic levels [20]. Statins are drugs that bind to albumin at a rate of 90–98%. Although hypoalbuminemia leads to an increase in the proportion of unbound drug, it can also accelerate drug metabolism and elimination, resulting in a net decrease in drug concentration [21].

A comprehensive study by Zeng et al. demonstrated that the lipid paradox observed in patients with acute myocardial infarction (the association of low LDL-C levels with high mortality) was present only in patients with high inflammatory risk (high-sensitivity C-reactive protein ≥ 3 mg/L), whereas this phenomenon was not observed in patients with a low inflammatory risk [13]. Yao et al. reported that low albumin levels in patients with coronary artery disease were associated with a high MACE risk and that this association interacted with the total cholesterol levels [12]. Lu et al. observed that the lipid paradox was present only in the population with a high malnutrition risk, implying that albumin functions as both a nutritional and an inflammatory marker [13,22]. Since albumin is a marker of both nutritional status and inflammation, patients with high nutritional risk are also at high inflammatory risk. The underlying mechanism of the lipid paradox in acute myocardial infarction remains unclear.

In our study, the association of the combination of low albumin and low baseline LDL-C with 95.7% hyporesponsiveness in the categorical analysis indicates that albumin is a clinically strong predictor and that low albumin may adversely affect the statin response. Regardless of the etiology of hypoalbuminemia, low albumin levels appear to be associated with statin hyporesponsiveness; however, further prospective studies are needed to elucidate the underlying mechanisms of this association.

Recently, two important studies conducted using the SWEDEHEART registry data have revealed the critical importance of the “strike early and strike strong” strategy [8,23]. Schubert et al. reported that achieving and maintaining the non-HDL target within the first 2 months after myocardial infarction was associated with the lowest cardiovascular event risk [8]. Leosdottir et al. reported that the 1-year MACE incidence was 1.79/100 patient-years in patients who received ezetimibe within the first 12 weeks after myocardial infarction, whereas this rate increased to 2.58/100 patient-years in patients who received ezetimibe after 12 weeks [23]. These findings reveal that the stepwise approach recommended by the current guidelines (statin monotherapy first, followed by addition of non-statin agents if the response is inadequate) inevitably leads to delays in treatment intensification and is associated with preventable cardiovascular events. The findings of this study provide a practical approach for risk stratification and treatment intensification in post-ACS patients. The combination of low baseline LDL-C and low albumin can be used as a simple and inexpensive tool to identify patients at very high risk for statin hyporesponsiveness (95.7%) in the early period. In these high-risk patients, combination therapy with statins and ezetimibe should be initiated proactively from discharge, instead of statin monotherapy.

### Limitations

This study has certain limitations. First, its retrospective design precluded the establishment of causality and potentially introduced selection bias. Second, the follow-up period was relatively short (21–28 d), and only the LDL-C response was evaluated; long-term cardiovascular outcomes (MACE, mortality) should be assessed in future studies. Third, statin adherence was not objectively measured, and a distinction between true hyporesponsiveness and non-adherence could not be made. Fourth, the loss of significance of albumin in multivariate analysis indicates collinearity between variables; however, the association of the combination of low albumin and low baseline LDL-C with 95.7% hyporesponsiveness in the categorical analysis demonstrates its clinical value. Fifth, genetic polymorphisms were not evaluated, and the effect of these factors on statin response should be investigated in future studies. Our study also included patients receiving atorvastatin monotherapy at doses of 40 mg or 80 mg as part of high-intensity statin therapy. Further investigation is needed to determine if similar findings are obtained with other statin types and doses. Finally, the proportion of female patients in the study population was low, necessitating validation of the findings in women. Despite these limitations, the findings of our study showed that the “strike early and strike strong” strategy after ACS should be applied to all patients but particularly as a priority to very high-risk patients (95.7% hyporesponsiveness) identified by the combination of low baseline LDL-C and low albumin, implying that the stepwise approach recommended in the current guidelines needs to be re-evaluated in this patient group.

## 5. Conclusions

In this study, hyporesponsiveness was observed in more than half of the patients receiving high-intensity statin therapy after ACS, and the combination of low LDL-C and low albumin at ACS onset significantly increased hyporesponsiveness. These two simple biochemical parameters are measured routinely and do not incur any additional cost. To avoid delays in intensified treatment after myocardial infarction, proactive combined LLT should be initiated from discharge in high-risk patients, instead of a stepwise approach. These simple biochemical markers can guide clinicians in identifying patients who would benefit the most from the “strike early and strike strong” strategy. Future studies should confirm these findings in prospective cohorts and evaluate the impact of early combined therapy strategies on long-term cardiovascular outcomes.

## Figures and Tables

**Table 1:** Demographic, clinical and laboratory characteristics of patients by response status.

	Response Status			
	Hyporesponder (*n* = 231)	Responder (*n* = 135)	Total (*n*= 366)	*p*-Value
Age, years	58.8 (10.87)	58.5 (10.97)	58.7 (10.89)	0.9042
Male, n (%)	200 (86.6%)	109 (80.7%)	309 (84.4%)	0.1372
Diagnosis				0.2022
STEMI, n (%)	136 (58.9%)	83 (61.5%)	219 (59.8%)	
Non-STEMI, n (%)	83 (35.9%)	50 (37.0%)	133 (36.3%)	
USAP, n (%)	12 (5.2%)	2 (1.5%)	14 (3.8%)	
DM, n (%)	91 (39.4%)	50 (37.0%)	141 (38.5%)	0.6548
HT, n (%)	87 (37.7%)	55 (40.7%)	142 (38.8%)	0.5598
Smoking Status				0.6782
Non-smoker, n (%)	61 (26.4%)	41 (30.4%)	102 (27.9%)	
Smoker, n (%)	142 (61.5%)	80 (59.3%)	222 (60.7%)	
Ex-smoker, n (%)	28 (12.1%)	14 (10.4%)	42 (11.5%)	
EF, %	50.3 (8.52)	50.0 (9.59)	50.2 (8.92)	0.9338
Glucose, mg/dL	152.0 (81.73)	148.3 (68.48)	150.6 (77.03)	0.9404
BUN, mg/dL	16.3 (5.85)	15.3 (4.57)	15.9 (5.43)	0.2647
Creatinine, mg/dL	1.0 (0.21)	1.0 (0.18)	1.0 (0.20)	0.5873
GFR, mL/min/1.73 m^2^	80.0 (17.54)	80.3 (16.18)	80.1 (17.03)	0.9579
Sodium, mmol/L	137.6 (3.06)	137.4 (3.11)	137.6 (3.08)	0.3769
ALT, U/L	30.0 (20.06)	28.6 (15.90)	29.5 (18.62)	0.9488
Albumin, g/L	41.7 (3.78)	43.3 (3.31)	42.3 (3.70)	0.0002
Uric Acid, mg/dL	5.6 (1.60)	5.5 (1.42)	5.6 (1.53)	0.7185
CRP, mg/L	15.1 (33.08)	10.3 (24.39)	13.3 (30.19)	0.9837
WBC, 10^3^/mm^3^	11.4 (3.66)	11.5 (3.61)	11.4 (3.63)	0.9975
HGB, g/dL	14.2 (1.89)	14.8 (1.72)	14.4 (1.85)	0.0016
NEU, 10^3^/mm^3^	8.3 (3.53)	8.1 (3.40)	8.2 (3.48)	0.7021
LYM, 10^3^/mm^3^	2.2 (1.12)	2.4 (1.14)	2.3 (1.13)	0.0931
Total Cholesterol, mg/dL	206.8 (49.90)	227.6 (50.43)	214.5 (51.03)	<0.0001
HDL-C, mg/dL	46.3 (11.10)	47.6 (11.18)	46.8 (11.13)	0.2814
VLDL-C, mg/dL	38.8 (36.87)	41.7 (35.27)	39.8 (36.26)	0.3759
Triglyceride, mg/dL	193.8 (184.33)	208.4 (176.33)	199.2 (181.31)	0.3749
LDL-C, mg/dL	126.6 (39.90)	147.3 (37.45)	134.2 (40.23)	<0.0001
Statin Dose, n (%)				0.3733
40 mg	222 (96.1%)	127 (94.1%)	349 (95.4%)	
80 mg	9 (3.9%)	8 (5.9%)	17 (4.6%)	

Chi-square *p*-value; Mann–Whitney U *p*-value; ALT, alanine transaminase; BUN, blood urea nitrogen; CRP, C-reactive protein; DM, diabetes mellitus; EF, ejection fraction; GFR, glomerular filtration rate; HGB, hemoglobin; HDL-C, high-density lipoprotein cholesterol; HT, hypertension; LDL-C, low-density lipoprotein cholesterol; LYM, lymphocyte; NEU, neutrophil; STEMI, ST-elevation myocardial infarction; USAP, unstable angina pectoris; VLDL-C, very low-density lipoprotein cholesterol; WBC, white blood cell.

**Table 2:** Relationship between LDL-C categories and albumin with hyporesponsiveness.

		Albumin Category				
LDL-C Category		<40	40–44	44<	Total	*p*-Value
<100		(*n* = 23)	(*n* = 31)	(*n* = 12)	(*n* = 66)	
	Response Status, n (%)					0.4521
	Hyporesponder	22 (95.7%)	27 (87.1%)	10 (83.3%)	59 (89.4%)	
	Responder	1 (4.3%)	4 (12.9%)	2 (16.7%)	7 (10.6%)	
	*p*-value	0.000	0.000	0.022	0.000	
100–129		(*n* = 30)	(*n* = 41)	(*n* = 25)	(*n* = 96)	
	Response Status, n (%)					0.0865
	Hyporesponder	24 (80.0%)	26 (63.4%)	13 (52.0%)	63 (65.6%)	
	Responder	6 (20.0%)	15 (36.6%)	12 (48.0%)	33 (34.4%)	
	*p*-value	0.001	0.088	0.845	0.002	
130–159		(*n* = 30)	(*n* = 56)	(*n* = 35)	(*n* = 121)	
	Response Status, n (%)					0.1147
	Hyporesponder	22 (73.3%)	29 (51.8%)	18 (51.4%)	69 (57.0%)	
	Responder	8 (26.7%)	27 (48.2%)	17 (48.6%)	52 (43.0%)	
	*p*-value	0.011	0.791	0.868	0.123	
≥160		(*n* = 6)	(*n* = 35)	(*n* = 42)	(*n* = 83)	
	Response Status, n (%)					0.9923
	Hyporesponder	3 (50.0%)	17 (48.6%)	20 (47.6%)	40 (48.2%)	
	Responder	3 (50.0%)	18 (51.4%)	22 (52.4%)	43 (51.8%)	
	*p*-value	1.000	0.868	0.761	0.744	

Chi-square *p*-value; LDL-C, low-density lipoprotein cholesterol.

**Table 3:** Results of the Cochran–Armitage trend analysis.

	Response Status			
	Hyporesponder (*n* = 231)	Responder (*n* = 135)	Total (*n* = 366)	*p*-Value
Albumin Category, n (%)				0.0004
<40	71 (30.7%)	18 (13.3%)	89 (24.3%)	
40–44	99 (42.9%)	64 (47.4%)	163 (44.5%)	
≥44	61 (26.4%)	53 (39.3%)	114 (31.1%)	
LDL-C Category, n (%)				<0.0001
<100	59 (25.5%)	7 (5.2%)	66 (18.0%)	
100–129	63 (27.3%)	33 (24.4%)	96 (26.2%)	
130–159	69 (29.9%)	52 (38.5%)	121 (33.1%)	
≥160	40 (17.3%)	43 (31.9%)	83 (22.7%)	

*p*-value for the Cochran–Armitage trend test.

**Table 4:** Measures of association.

	Albumin				LDL-C			
	Value	ASE	Lower 95% CI	Upper 95% CI	Value	ASE	Lower 95% CI	Upper 95% CI
Somers’ D C|R	0.2171	0.0552	0.1089	0.3253	0.3086	0.0547	0.2013	0.4159
Somers’ D R|C	0.1566	0.0398	0.0785	0.2346	0.1947	0.0347	0.1267	0.2627
Pearson Correlation	0.1968	0.0491	0.1005	0.2931	0.2730	0.0464	0.1821	0.3639
Spearman Correlation	0.1948	0.0495	0.0978	0.2918	0.2677	0.0475	0.1745	0.3608

ASE, average square error; CI, confidence interval; LDL-C, low-density lipoprotein cholesterol.

**Table 5:** Univariate and multivariate logistic regression analysis for the predictors of response status (hyporesponder or responder).

	Response Status = Responder			
	Univariate Analysis		Multivariate Analysis	
Covariate	Odds Ratio (95% CI)	OR *p*-Value	Odds Ratio (95% CI)	OR *p*-Value
Age	1.0 (1.0–1.0)	0.8151		
Sex (Female)	1.5 (0.9–2.7)	0.1401		
MI Type				
non-STEMI	1.0 (0.6–1.5)	0.9542		
USAP	0.3 (0.1–1.3)	0.0960		
DM	1.1 (0.7–1.7)	0.6563		
HT	0.9 (0.6–1.4)	0.5612		
Smoking Status				
Ex-smoker	0.9 (0.4–1.8)	0.7380		
Non-smoker	1.2 (0.7–1.9)	0.4743		
EF	1.0 (1.0–1.0)	0.7554		
Glucose	1.0 (1.0–1.0)	0.6430		
BUN	1.0 (0.9–1.0)	0.0660		
Creatinine	0.6 (0.2–1.7)	0.3621		
GFR	1.0 (1.0–1.0)	0.8971		
Sodium	1.0 (0.9–1.0)	0.4652		
ALT	1.0 (1.0–1.0)	0.4841		
Albumin	1.1 (1.1–1.2)	0.0001	1.1 (1.0–1.2)	0.0191
Uric Acid	1.0 (0.9–1.1)	0.9070		
WBC	1.0 (0.9–1.1)	0.9403		
HGB	1.2 (1.1–1.4)	0.0031	1.1 (1.0–1.3)	0.0822
NEU	1.0 (0.9–1.1)	0.7020		
LYM	1.2 (1.0–1.4)	0.1332		
Total Cholesterol	1.0 (1.0–1.0)	0.0001	1.0 (1.0–1.0)	0.1410
HDL-C	1.0 (1.0–1.0)	0.2881		
LDL-C	1.0 (1.0–1.0)	0.0001	1.0 (1.0–1.0)	0.0041
VLDL-C	1.0 (1.0–1.0)	0.4714		
Triglycerides	1.0 (1.0–1.0)	0.4693		
C-Reactive Protein	1.0 (1.0–1.0)	0.2432		
Statin Dose (40 mg)	0.6 (0.2–1.7)	0.3780		

ALT, alanine transaminase; BUN, blood urea nitrogen; CI, confidence interval; DM, diabetes mellitus; EF, ejection fraction; GFR, glomerular filtration rate; HGB, hemoglobin; HDL-C, high-density lipoprotein-cholesterol; HT, hypertension; LDL-C, low-density lipoprotein-cholesterol; LYM, lymphocyte; MI, myocardial infarction; NEU, neutrophil; OR, odds ratio; STEMI, ST-elevation myocardial infarction; USAP, unstable angina pectoris; VLDL-C, very low-density lipoprotein cholesterol; WBC, white blood cell.

**Table 6:** ROC Analysis Results for Albumin, LDL-C, and LDL-C + Albumin.

	AUC	SE	95% CI		*p*	Cut Off	Sensitivity	Specificity
Albumin	0.618	0.030	0.559	0.676	0.0001	41.15	0.776	0.424
LDL-C	0.652	0.029	0.596	0.708	0.0001	128.50	0.719	0.519
LDL-C + albumin	0.670	0.028	0.615	0.725	0.0001	5292.78	0.709	0.515

AUC, area under the curve; CI, confidence interval; LDL-C, low-density lipoprotein cholesterol; ROC, receiver operating characteristic; SE, standard error.

**Table 7:** Pair-wise comparison of ROC curves.

	Albumin	LDL-C + Albumin	LDL-C
Albumin	-	*p* = 0.0743	*p* = 0.3435
LDL-C + albumin		-	*p* = 0.0182
LDL-C			-

Note: (-) indicates that comparison between the same variables is not applicable.

## Data Availability

The data presented in this study are available on request from the corresponding author.
